# The Importance of the Assessment of Epicardial Adipose Tissue in Scientific Research

**DOI:** 10.3390/jcm11195621

**Published:** 2022-09-23

**Authors:** Przemysław Cheładze, Adrian Martuszewski, Rafał Poręba, Paweł Gać

**Affiliations:** 1 Centre for Diagnostic Imaging, 4th Military Hospital, Weigla 5, PL 50-981 Wroclaw, Poland; 2 Department of Population Health, Division of Environmental Health and Occupational Medicine, Wroclaw Medical University, Mikulicza-Radeckiego 7, PL 50-368 Wroclaw, Poland; 3 Department of Internal and Occupational Diseases, Hypertension and Clinical Oncology, Wroclaw Medical University, Borowska 213, PL 50-556 Wroclaw, Poland

**Keywords:** epicardial adipose tissue, diagnostic imaging, scientific research

## Abstract

Epicardial adipose tissue (EAT) exhibits morphological similarities with pericardial adipose tissue, however, it has different embryological origin and vascularization. EAT is a metabolically active organ and a major source of anti-inflammatory and proinflammatory adipokines, which have a significant impact on cardiac function and morphology. Moreover, it can regulate vascular tone by releasing various molecules. The relationship between EAT and cardiovascular disease and diseases of other organ systems is now considered a common discussion subject. The present clinical review article summarizes the epidemiological findings based on imaging techniques in studies conducted so far. In conclusion, evaluation of the epicardial adipose tissue constitutes a helpful scientific parameter, which can be assessed by means of different diagnostic imaging examinations.

## 1. Introduction

Fatty tissue is a complex organ in the body, that performs numerous functions—local and systemic. The fact that adipose tissue not only has a thermal insulation function and the function of an energy store (in the form of triglycerols) has been known for over 30 years. Many studies have shown that it affects, among others, the immune system, affects the renin-angiotensin-aldosterone system and the wall of blood vessels and cooperates with the autonomic nervous system [1,2]. Fatty tissue is very often referred to as an organ, although it does not meet the classical definition. It is indicated that this is an important endocrine organ [3]. Adipocytes (the cells that make up fat) release several substances—adipokines (also called adipocytokines). Each change in the extent of their secretion is associated with specific consequences, mainly with the development of specific diseases. This applies, among others, to cardiovascular diseases, insulin resistance, hypertension, heart rhythm disorders, and heart failure [4,5,6,7].

According to the literature, the correct content of adipose tissue in men is 12–20%, while in women its correct range is slightly higher—it is between 20% and 30%. According to the definition of the World Health Organization (WHO), obesity is an excess of body fat—in men ≥25%, and in women ≥35%. First, it accumulates in the thighs, hips, and buttocks in women, while in men, it accumulates mainly in the abdominal area. The remaining adipose tissue is the visceral deposit, the retroperitoneal deposit, and the deposit located within the genitals. A positive caloric balance (excessive energy intake with a low level of energy expenditure) results in an increase in the size of adipocytes. The main reason for this mechanism is the accumulation of fatty acids in these cells, which also leads to the structural reconstruction of fat cells and constitutes a signal that triggers an inflammatory response [8,9].

Dedicated devices are usually used to calculate body fat. The most popular is bioelectrical impedance analysis (BIA). Selected parameters of adipose tissue, especially in specific parts of the body, can also be analyzed using diagnostic imaging methods [2].

## 2. Cardiac Adipose Tissue

**Figure 1:**
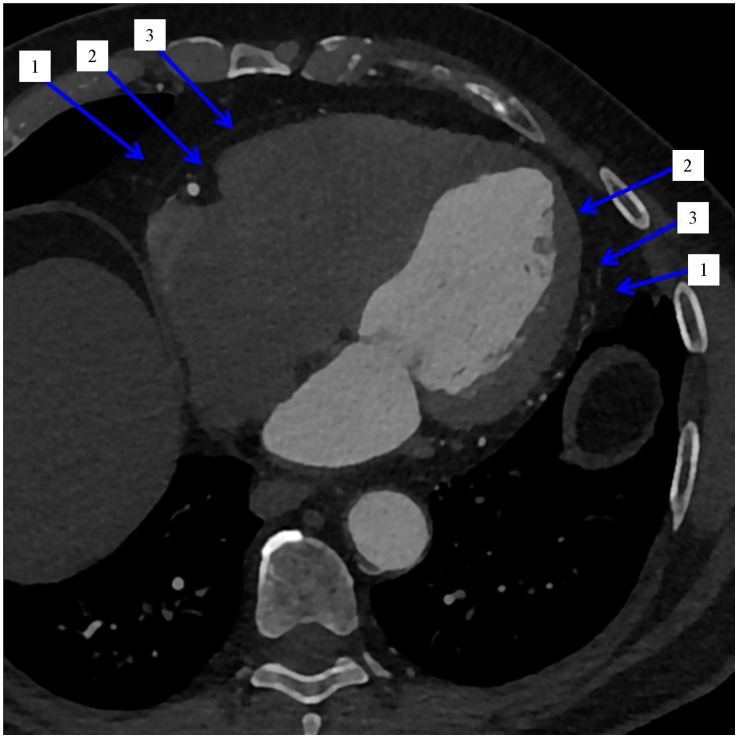
Cardiac computed tomography. Axial reconstruction. Blue arrows indicate: 1. Pericardial adipose tissue. 2. Epicardial adipose tissue. 3. Pericardium.

**Figure 2:**
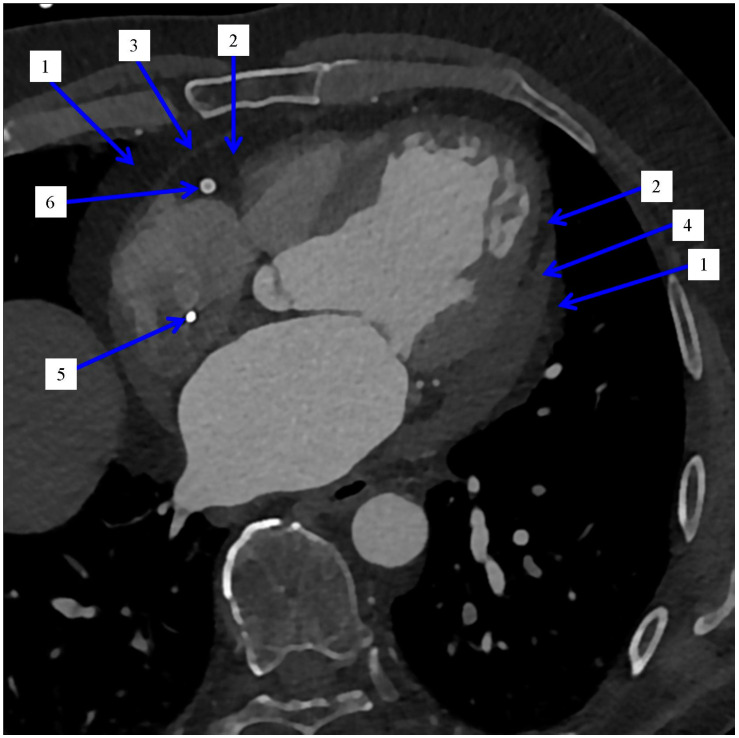
Cardiac computed tomography in a patient after interventional treatment. Multiplanar oblique reconstruction. Blue arrows indicate: 1. Pericardial adipose tissue. 2. Epicardial adipose tissue. 3. Pericardium. 4. Fluid in the pericardium. 5. Cardiac pacemaker electrode in the right atrium. 6. Right coronary artery stent.

The correlation of visceral adipose tissue with the development of many diseases aroused wide interest in scientific circles. The so-called “fattening” of the heart muscle pathomorphologists was already interested in the 20th century [10]. In 1955, the first data on the measurement of adipose tissue were published [11]. Initially, the focus was on visceral adipose tissue (VAT), but over time, the main subject of the analysis was (and remains to this day) visceral adipose tissue located on the surface of the heart—i.e., epicardial adipose tissue (EAT). Together with pericardial adipose tissue (PeAT), it forms cardiac adipose tissue (CAT), Figure 1 and Figure 2.

There is a dilemma of different nomenclature used in the literature on heart adipose tissue. The following types of adipose tissue can be distinguished: Epicardial adipose tissue (EAT), pericardial adipose tissue (PeAT), and paracardial adipose tissue (PaAT). However, EAT can be expressed as thickness (EAT-t) and volume (EAT-v) [12]. Many researchers measure EAT using computed tomography (CT) or transthoracic echocardiography (ECHO), but magnetic resonance imaging (MRI) remains the gold standard for this purpose due to its good spatial resolution [13]. ECHO is widely available and relatively costless, but obtained images are not as reproducible as in CT or MRI.

### 2.1. Distribution of the Epicardial Adipose Tissue

The pericardial adipose tissue, like typical locoregional adipose tissue, develops from the mesoderm. PeAT is highly vascularized through the arterial branches of the mediastinum. The epicardial adipose tissue develops from the visceral leaf of the extramedullary mesoderm, its cells migrate to the surface of the heart. Coronary artery branches provide EAT vascularity. In the literature, it is often possible to find a division of EAT into two subgroups—muscle and perinodullary. In humans, EAT covers a significant part of the heart surface. According to various sources, this applies from 56% to 100%, with an average of 80%. Its clusters are located primarily in the interventricular and atrioventricular grooves. In addition, it also occurs on the course of coronary vessels, along the right edge of the wall of the right ventricle, on the anterior wall, and around the tip of the heart. Most often, the EAT weight is about 20% of the heart weight. Many authors report that in women, EAT represents a slightly higher percentage of heart weight than in men. Others, on the other hand, do not see such a dependence depending on gender. There are also reports on the influence of age on the thickness of this type of adipose tissue, which is also a questionable issue [1,14,15]. The EAT index proposed by Shmilovich et al. [16] does not consider age or gender. The volume of adipose tissue is expressed in relation to the body surface area. The main value of this index is 95 percentiles (68.1 cm^3^/m^2^)—each higher value increases the risk of cardiovascular diseases.

A different ratio of EAT to myocardium is observed depending on the ventricle. In the case of the right ventricle (RV), it is much larger than for the left ventricle (LV). It is 0.48 for RV and 0.15 for LV in males. However, in females, it is 0.61 for RV and 0.17 for LV. It is indicated that the mass of EAT, and the mass of myocardium is characterized by a linear relationship—the proportions between these two elements do not change either because of hypertrophy or myocardial ischemia [1]. There are also reports that in exceptional cases, the high thickness of epicardial fat may interfere with the mechanics of the left ventricle [17].

The volume of EAT increases with the amount of intra-abdominal visceral tissue. Therefore, the measurement of epicardial fat is an important VAT mass index [18].

### 2.2. Imaging and Measurement Methods of Epicardial Adipose Tissue

The measurement of epicardial adipose tissue is carried out primarily by ECHO. It is a widely available, fast, and safe method. The thickness of adipose tissue measured by this technique correlates well with its volume obtained in the measurement using CT [1,19]. The disadvantage of this technique, especially in the case of two-dimensional ECHO, is the lower quality of the measurement—in the test, the amount of EAT is obtained based on thickness measurement, not volume, as in the case of MRI or CT. Three-dimensional ECHO is much better, but its availability is much lower, and sometimes it is a time-consuming method. In the examination, the right ventricle of the heart is analyzed, and EAT measurement is performed in cardiac diastole—during three consecutive cycles of this organ. Thanks to this, it is possible to compare the obtained result with MRI and CT measurements. Some recommendations allow measurements to be made during cardiac contraction—however, it is not possible to compare the results obtained with measurements made by other methods. It is also repeatedly pointed out that the measurement in echocardiography may be unreliable due to the need to identify the pericardium—this is technically difficult in most cases and additionally complicated in obese people (obstacles in obtaining superior quality images) [20,21,22].

Even though EAT measurement is most often performed using ECHO, the “gold standard” is the cardiac MRI method. In MRI, a particularly good spatial resolution is obtained. This is the only imaging study that allows ex vivo measurement of the volume of epicardial fat. Among the advantages, there is no need for radiation and contrast agents. Defects include prohibitive cost of testing, lower availability, and considerable time requirements [20,23,24].

CT of the chest also allows for EAT measurement. In general, this technique is characterized by good spatial resolution, repeatability, and the possibility of volume measurement. The main disadvantage is exposure to ionizing radiation and the use of contrast agents with iodine [20]. The use of two-dimensional computed tomography to measure epicardial fat is troublesome and reduces its accuracy. It requires a manual calculation of the area of fat tissue in each of the analyzed sections. The next step is to multiply the fields by the thickness of individual scans [25]. Therefore, it is preferable to use automatic analysis of three-dimensional images. This method is faster, less labor-intensive, and above all, more accurate [26].

In the opinion of the authors, the implementation of routine EAT assessment in the analysis of computed tomography images should be considered analogous to the routine assessment of, e.g., coronary artery calcium score. It is postulated to encourage physicians to measure EAT in their cohorts.

## 3. Epicardial Adipose Tissue in CLINICAL Medicine

Studies to date have shown an association between EAT and cardiovascular diseases, as well as an association between EAT and non-cardiovascular systemic diseases.

Recently, it has been proposed that cardiovascular fat is a marker of the risk of cardiovascular disease (CVD) [20,27]. Numerous publications indicate that excess epicardial fat plays a significant role in the development of this group of diseases, but this issue remains insufficiently understood, even though it may be an important parameter of risk assessment [28].

Substantial amounts of EAT correlate with the occurrence of the coronary syndrome, weakening of atherosclerotic plaques, or atrial fibrillation [29,30,31,32,33]. It was observed that the increased amount of EAT causes atherosclerotic plaques in the arteries to be of a more dangerous nature. For this reason, it is believed that the thickness of the epicardial fat is important for the development of coronary atherosclerosis [34]. This boils down to a conclusion indicating a higher risk of platelet formation in coronary arteries depending on EAT thickening [35,36]. It has been shown that the thickness of the epicardial fat is the only independent factor of slowed coronary flow [37]. The formation of atherosclerotic plaques is easier when the coronary arteries are in direct contact with the epicardial fat. This is associated with the continuous adverse effect of pro-inflammatory cytokines. It has also been proven that the larger the volume of EAT, the smaller the diameter of the coronary arteries. Atherosclerotic plaque, which is in the vicinity of epicardial fat tissue, is most often characterized by lower calcification and greater instability. This, in turn, results in an increased risk of its rupture, which then results in the occurrence of the acute coronary syndrome [38,39]. In the study by Bertaso et al. [40], an independent relationship was observed between EAT and cardiovascular risk factors, coronary artery calcification, and carotid artery stenosis. In addition, it was also shown that the volume of epicardial fat (especially this parameter in relation to the left atrioventricular sulcus) is an independent predictor of cardiovascular diseases in patients with diagnosed type 2 diabetes without a history of coronary artery disease [41]. The literature on the subject indicates that the volume of epicardial adipose tissue above 300 cm^3^ correlates with a 4-fold higher risk of developing atherosclerotic changes in coronary vessels. This parameter is currently the most sensitive one possible in this respect [42].

This is also confirmed by a large study conducted on the German population, where the study group included 4093 people. So far, the respondents have not suffered from any cardiovascular diseases. The amount of epicardial fat was assessed by computed tomography. A wide range of values (12.99 mL to 390.0 mL) was noted, and the median volume was 85.9 mL. The whole group was divided into four subgroups, depending on the EAT volume quartiles. Each subsequent quartile was associated with more classic risk factors for CVD (hypertension, greater waist circumference, dyslipidemia, diabetes). All subjects were followed for eight years for coronary events and for the need for hospitalization (heart disease or death). During this period, they were confirmed in 130 patients, and their frequency was directly proportional to the subsequent qualifying quartiles. People who were qualified in the 4th quartile had a five times higher risk of a coronary incident compared to people in the 1st quartile. At the same time, an increase in the amount of epicardial fat was observed. Doubling its volume increased the risk of a coronary incident by 1.5–2.24 times in correlation with the baseline values [43].

Cardiovascular diseases very often coexist with chronic obstructive pulmonary disease (COPD). The subject of research, although so far few, is also the relationship between COPD and epicardial adipose tissue [20,44]. Zagaceta et al. [45] indicate that the presence of this disease is a statistically significant predictor of EAT volume. The authors assessed the volume of EAT using CT. Moreover, Demir et al. [46] and Kiraz et al. [47] confirmed that in people with COPD, the layer of epicardial fat is larger. The measurement was conducted this time using the echocardiographic method. However, there are also analyses available, e.g., Kaplan et al. [22], which state that in people with COPD, epicardial fat is thin.

A higher volume of epicardial fat increases the risk of cardiovascular disease in people with a negative history of cardiovascular problems. This aspect may indicate a strong correlation between COPD and cardiovascular diseases [48]. Some studies confirm that EAT is of key importance in systemic inflammation in COPD [49,50]. Fatty tissue dysfunctions, including severe inflammation, may be a trigger for subclinical systemic inflammation. This phenomenon is very often observed in patients with obstructive pulmonary disease. The volume of visceral adipose tissue correlates, among other things, with mortality due to cardiovascular diseases or high serum levels of interleukin 6 (IL-6). However, it has not yet been shown whether respiratory impairment, in turn, increases the accumulation of visceral adipose tissue [23].

An increased amount of epicardial fat was also observed in people with thyroid disease. This concerned, among others, subclinical hypothyroidism, which negatively affects the cardiovascular system [51]. Sayin et al. [52] analyzed whether the amount of EAT can be reduced in this group of patients. They measured twice using ECHO. It has been shown that treatment with L-thyroxine, resulting in the restoration of euthyreosis, results in a reduction in the amount of epicardial adipose. This is also confirmed by the study conducted by Korkmaz et al. [53]. In addition, the authors found a significant correlation between EAT and TSH [51]. A study conducted by Asik et al. [54] proved that the thickness of epicardial fat in people with Hashimoto thyroiditis and in patients with hypothyroidism (subclinical and overt) can be a useful indicator of early atherosclerosis. In the literature, however, one can find publications that contradict this assumption, including Yazici et al. [55] or Santos et al. [56].

An increase in fat mass is also observed in patients with non-alcoholic fatty liver disease (NAFLD) [57]. The relationship between the thickness of EAT and the stage of NAFLD and the risk of cardiovascular diseases is indicated [58]. In another study [59], the correlation of epicardial adipose tissue with the severity of steatosis, fibrosis, and the occurrence of cardiovascular diseases in the group of patients with NAFLD was proven. Increased EAT volume (measured by echocardiography) is also associated with the stage of steatosis and fibrosis in the liver [60].

### 3.1. Epicardial Adipose Tissue and Atrial Fibrillation Recurrence

In the literature, one can come across a publication on the proportional correlation between the amount of EAT and an increased risk of recurrent atrial fibrillation after catheter ablation. Atrial fibrillation (AF) is the most common arrhythmia worldwide. Several studies report that increased EAT volume is associated with a higher risk of recurrence of AF after catheter ablation due to slowing and disturbance of conduction by an increased amount of adipose tissue. After analyzing a group of 1840 patients with AF, a significant correlation was found between the amount of epicardial adipose tissue and an increased risk of recurrent atrial fibrillation after catheter ablation. It has been shown that patients with an average age of less than 60 years have a higher risk of AF recurrence due to more EAT. The results suggest that younger patients are at greater risk of recurring atrial fibrillation after catheter ablation. The literature summarizes that there are EAT parameters (total volume, peri-atrial volume, and ratio of the periatrial to total EAT volume) that are of greater importance in predicting the risk of recurrence of AF following catheter ablation. The work concluded that the volume of EAT should be included in the risk assessment of recurrent atrial fibrillation before the patient undergoes catheter ablation [61,62,63,64,65].

### 3.2. Epicardial Adipose Tissue and Relation to Metabolism in Old Patients

In a study by Conte et al. [66] has been observed correlation between aging and an increase in EAT volume. The complex phenomenon of aging is accompanied by deterioration of biological functions, decreased metabolism, and greater susceptibility to inflammation. Age, especially when it is advanced, is one of the risk factors for major chronic human diseases. Low-grade inflammation promotes cardiovascular disease in the elderly. It does so by increasing the risk of insulin resistance and atherosclerosis. The increase in EAT, i.e., metabolically active tissue, is associated with greater production and secretion of pro-inflammatory mediators. Increased production of the previously mentioned causes the progression of cardiovascular diseases by acting on the heart muscle and coronary vessels [66]. According to the literature, in people over 65 years of age, the mean EAT volume was 22% higher than in younger subjects, which suggests that the volume of epicardial adipose tissue increases with age. Interestingly, it was found that the increase in EAT volume is more strongly related to age than BMI or waist circumference [67]. A study of 120 people was conducted to investigate the correlation between anthropometric values and EAT in older and younger people. After analyzing the results, a correlation was found between the increased volume of EAT and age, waist circumference, and thigh circumference. A correlation was observed between EAT and fasting insulin and insulin resistance only in the elderly group. The study highlights the importance of epicardial fat in estimating cardiometabolic risk in elderly patients, especially the elderly [68]. Other authors emphasize the relationship between fatty liver, the amount of visceral fat, and the occurrence of metabolic syndrome in the elderly. Comparing the group of patients with the metabolic syndrome to the group without the metabolic syndrome, it was concluded that the amount of EAT is significantly higher in the first group. A correlation between the increased volume of EAT and the occurrence of the metabolic syndrome has been observed by Stramaglia et al. [69].

## 4. Clinical Practice and Studies Focused on Heart Adipose

Measurement of epicardial adipose tissue is non-invasive but provides some information about cardiovascular health. To date, various studies about heart adipose tissue have been conducted. Studies that were mentioned in the current review and original articles are summarized in Table 1.

## 5. Epicardial Adipose Tissue and COVID-19-Related Cardiac Syndrome

The COVID-19 (disease caused by SARS-CoV-2) pandemic since the fall of 2019 has been a challenge for modern medicine. The conducted research on the importance of epicardial adipose tissue in patients with COVID-19 has shown so far that higher EAT volume and lower EAT density may be independent predictors of both an unfavorable course of the disease, including death, as well as cardiovascular complications COVID-19 [86,87,88]. For example, the COVID mortality associated with cardiovascular calcifications in COVID-19 can also be explained by epicardial adipose tissue [88]. Moreover, some cardiovascular complications are asymptomatic during acute SARS-CoV-2 infection, but emerging data have reported on post-COVID-19 heart syndrome. It has been suggested that high EAT volume and low EAT density in computed tomography may indicate myocardial injury in COVID-19 patients [89]. The importance of EAT in this group of patients may be explained by the immunomodulatory properties of EAT, because of which EAT may constitute a tissue reservoir for SARS-CoV-2 [90].

## 6. Summary

**Figure 3:**
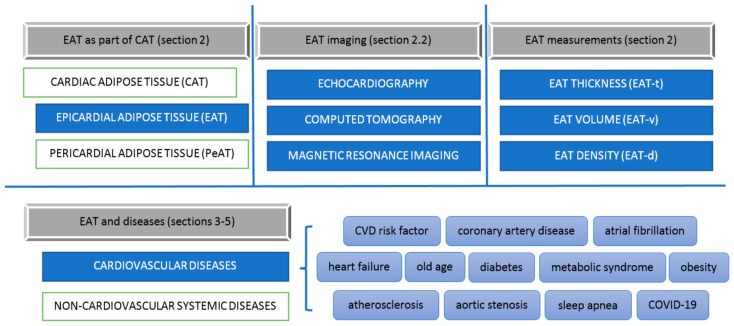
Epicardial adipose tissue—resume.

According to numerous scientific studies, epicardial adipose tissue, as a metabolically active reservoir of visceral adipose tissue, plays a key role in the pathogenesis of many diseases, Figure 3.

Epicardial adipose tissue can be considered both a risk factor for cardiovascular disease and a marker of cardiovascular disease. Therefore, measuring the volume of epicardial adipose tissue is crucial in assessing the relationship between epicardial adipose tissue and various pathologies. Measurements of the thickness and density of epicardial adipose tissue are of less importance [33,70,71,72,73]. One of the latest studies in this field proves explicitly that the thickness of epicardial adipose tissue is not helpful in predicting cardiovascular adverse events. The authors observed this in patients not undergoing hemodialysis. In turn, it was shown that increased EAT thickness is significantly associated with older age, female gender, low level of hemoglobin, and low early diastolic velocity of the mitral ring [74].

Reducing its volume is, therefore, a prominent issue in the prevention and treatment of many diseases. One of the most effective therapeutic methods is weight loss, which causes a parallel decrease in the volume of adipose tissue that is located around the heart. This method works in all people—in the general population, patients with metabolic syndrome, and patients with overweight or obesity [75,76]. Another technique, much more invasive, is surgery. It usually includes bariatric surgery [77,78]. So far, the only group in which surgery in this area is ineffective has proved to be people with diagnosed obstructive sleep apnea [79]. However, weight loss for many people is difficult or unsuccessful, which further discourages them. Therefore, pharmacotherapy is used to reduce the volume of epicardial fat. Long-term use of statins significantly reduces the volume of EAT [80,81,91]. In one of the studies, a decrease of 16.2% was observed in people who underwent pharmacological therapy for a period of 1.2 years. The best known and tested drug in this respect is atorvastatin, e.g., at a dose of 80 mg/day for a year [92,93,94]. Its effectiveness has been demonstrated, among others, in postmenopausal women [82] and patients undergoing pulmonary venous isolation due to atrial fibrillation [83]. Other pharmacological possibilities, characterized by effects on endocardial fat tissue, concern the use of metformin, thiazolidinedione, SGLT2 inhibitors, GLP-1 agonists, DPP-4 inhibitors, canakinumab, methotrexate, colchicine [84,95,96]. Multidirectional treatment, as always, turns out to be the most effective, even in people with diagnosed comorbidities, e.g., diabetes. Hence, a significant reduction in EAT can be achieved through weight reduction, undertaking physical activity, diet modification, and pharmacological treatment, and ultimately also through surgical intervention [85,97].

## Figures and Tables

**Table 1:** Studies focused on heart adipose tissue.

Refs.	Imaging System	Type of Adipose Tissue	Context of Analyzing Adipose Tissue (Most Important, Based on the Aim of the Study and Conclusions)	Study Population Size
Gaborit et al. [6]	MRI	EAT-v	Metabolic risk factors, coronary artery disease	63
Shmilovich et al. [16]	Non-contrast CT	EAT-v	Predicting major adverse cardiovascular events	516
Mahabadi et al. [21]	Non-contrast CT	EAT-v	Left atrial size, prevalent and incident atrial fibrillation	3467
Kaplan et al. [22]	ECHO	EAT-t	Chronic obstructive pulmonary disease, right ventricular systolic dysfunction	138 (included 40 control subjects)
Mahajan et al. [23]	MRI	PeAT	Animal, autopsy pericardial adipose measurements	10
Saremi et al. [25]	Contrast CT	EAT-v	Regions of heart adipose pockets, comparison with EAT-t	60
Park et al. [26]	Contrast CT	EAT-v	Threshold-based 3D segmentation, coronary CT angiography	100 (included 40 control subjects)
Ito et al. [29]	Contrast CT	EAT-v	Coronary plaque vulnerability, acute coronary syndrome	117
Yerramasu et al. [30]	Non-contrast CT	EAT-v	Metabolic syndrome, coronary artery calcium burden, diabetes mellitus	333
Picard et al. [31]	Contrast CT	EAT-t	Coronary artery disease	970
Nakanishi et al. [32]	Contrast CT	EAT-v	Coronary artery disease, acute coronary syndrome	517
Okada et al. [34]	Contrast CT	EAT-v	Coronary artery disease	140
Demircelik et al. [35]	Contrast CT	EAT-t	Coronary artery disease	131
Yamashita et al. [36]	Contrast CT	EAT-v	Non-culprit coronary lesions, coronary plaque burden	54
Alexopoulos et al. [39]	Contrast CT	EAT-v	Coronary artery disease	214
Uygur et al. [41]	Contrast CT	EAT-v	Coronary artery disease, diabetes mellitus	157
Greif et al. [42]	CT	PeAT	Coronary artery disease, intermediate pretest likelihood	286
Janik et al. [43]	Non-contrast CT	EAT-v	Coronary artery disease, intermediate pretest likelihood, ischemic heart disease	97
Kalaycioglu et al. [44]	ECHO	EAT-t	Chronic obstructive pulmonary disease, systolic pulmonary arterial pressure	129
Zagaceta et al. [45]	CT	EAT-v	Chronic obstructive pulmonary disease, smoking history, physical activity	241
Demir et al. [46]	ECHO	EAT-t	Chronic obstructive pulmonary disease, metabolic syndrome, ischemic heart disease	166 (included 84 control subjects)
Kiraz et al. [47]	ECHO	EAT-t	Chronic obstructive pulmonary disease, BODE index	202 (included 45 control subjects)
Ding et al. [48]	CT	PeAT	Coronary artery disease	998
Unubol et al. [51]	ECHO	EAT-t	Subclinical hypothyroidism	62 (included 25 control subjects)
Sayin et al. [52]	ECHO	EAT-t	Subclinical hypothyroidism	86 (included 42 control subjects)
Korkmaz et al. [53]	ECHO	EAT-t	Subclinical hypothyroidism	85 (included 24 control subjects)
Asik et al. [54]	ECHO	EAT-t	Carotid intima media thickness, Hashimoto thyroiditis, subclinical hypothyroidism	57
Yazıcı et al. [55]	ECHO	EAT-t	Carotid intima media thickness, subclinical hypothyroidism, restoration of the euthyroid state	73 (included 30 control subjects)
Santos et al. [56]	ECHO	EAT-t	Subclinical hypothyroidism	100 (included 48 control subjects)
Canpolat et al. [62]	ECHO	EAT-t	Atrial fibrillation, ablation	234
Chao et al. [63]	ECHO	EAT-t	Atrial fibrillation, ablation	283
Sanghai et al. [64]	Contrast CT	EAT-v	Indexed left atrial epicardial adipose tissue (iLAEAT), atrial fibrillation, ablation	274
Kawasaki et al. [65]	Contrast CT	EAT-v	Atrial fibrillation, ablation, cardiac sympathetic nerve activity	64
Guglielmi et al. [67]	MRI	EAT-v	Expansion of intermuscular adipose tissue, sedentary subjects	32
Karadag et al. [68]	ECHO	EAT-t	Metabolic syndrome, visceral adiposity	120
Stramaglia et al. [69]	ECHO	EAT-t	Metabolic syndrome, visceral adiposity, hepatic steatosis, risk of malnutrition in the obese elderly	55
Hell et al. [70]	Non-contrast CT	EAT-v	Epicardial adipose density, pre-test probability, coronary artery disease, SPECT	213
Goeller et al. [71]	Non-contrast CT	EAT-v	Epicardial adipose density, early atherosclerosis, plaque inflammation, major adverse cardiac events, coronary calcium	456
Nerlekar et al. [72]	Contrast CT	EAT-v	Epicardial adipose tissue density, non-obstructive coronary artery disease, statin therapy	90
Kataoka et al. [73]	CT	EAT-v	Coronary artery spasm, total abdominal adipose tissue area, abdominal visceral adipose tissue	110
Chen et al. [74]	ECHO	EAT-t	Hemodialysis patients, adverse cardiovascular events	189
Nakazato et al. [75]	Non-contrast CT	EAT-v	Weight change, coronary calcium score	374
Fu et al. [76]	MRI	EAT-t	Weight change, metabolic syndrome, diabetes mellitus	57 (included 25 control subjects)
Willens et al. [77]	ECHO	EAT-t	Bariatric surgery, metabolic syndrome, abdominal visceral adipose tissue	23
Kim et al. [78]	ECHO	EAT-t	Effects of exercise training, ventricular epicardial adipose thickness	24
Gaborit et al. [79]	MRI	EAT-v	Sleep apnea, bariatric surgery, morbid obesity	23
Parisi et al. [80]	ECHO	EAT-t	Statin therapy, aortic stenosis, cardiac surgery	193
Raggi et al. [81]	CT	EAT-v	Epicardial adipose tissue attenuation, statin therapy, coronary artery calcium score, postmenopausal women	420
Alexopoulos et al. [82]	Non-contrast CT	EAT-v	Electron beam CT scans, statin therapy, postmenopausal women	420
Soucek et al. [83]	Contrast CT	EAT-v	Statin therapy, atrial fibrillation, pulmonary vein isolation	79
Bouchi et al. [84]	MRI	EAT-v	Luseogliflozin therapy, diabetes mellitus	19
Cosson et al. [85]	Non-contrast CT	EAT-v	Coronary artery calcification, diabetes mellitus	409

CT, computed tomography; ECHO, echocardiography; EAT-t, epicardial adipose tissue thickness; EAT-v, epicardial adipose tissue volume; MRI, magnetic resonance imaging; PaAT, paracardial adipose tissue; PeAT, pericardial adipose tissue.

## Data Availability

Not applicable.

## References

[1] Toczyłowski K., Gruca M., Baranowski M. (2013). Epicardial Adipose Tissue and Its Role in Cardiac Physiology and Disease. Postępy Hig. Med. Dośw..

[2] Murawska-Ciałowicz E. (2017). Adipose Tissue–Morphological and Biochemical Characteristic of Different Depots. Postępy Hig. Med. Dośw..

[3] Cinti S. (2005). The Adipose Organ. Prostaglandins Leukot. Essent. Fat. Acids.

[4] Sperling M., Grzelak T., Czyżewska K. (2016). Endocrine function of adipose tissue in historical perspective. Hygeia Public Health.

[5] de Feyter P.J. (2011). Epicardial Adipose Tissue: An Emerging Role for the Development of Coronary Atherosclerosis. Clin. Cardiol..

[6] Gaborit B., Kober F., Jacquier A., Moro P.J., Cuisset T., Boullu S., Dadoun F., Alessi M.-C., Morange P., Clément K. (2012). Assessment of Epicardial Fat Volume and Myocardial Triglyceride Content in Severely Obese Subjects: Relationship to Metabolic Profile, Cardiac Function and Visceral Fat. Int. J. Obes..

[7] Iozzo P. (2011). Myocardial, Perivascular, and Epicardial Fat. Diabetes Care.

[8] Kłoda K., Mierzecki A. (2021). The Less Adipose Tissue the Better?. Lek. POZ.

[9] Stupnicki R., Tomaszewski P. (2016). Body mass index and body fat content in adults. Hygeia Public Health.

[10] Filipiak J.K. (2015). The commentary. The epicardial adipose tissue—what we know about the role of statins in reducing it?. Chor. Serca Naczyń.

[11] Reiner L., Mazzoleni A., Rodriguez F.L. (1955). Statistical Analysis of the Epicardial Fat Weight in Human Hearts. AMA Arch. Pathol..

[12] Zhou H., An D.-A., Ni Z., Xu J., Zhou Y., Fang W., Lu R., Ying L., Huang J., Yao Q. (2022). Magnetic Resonance Imaging Quantification of Accumulation of Epicardial Adipose Tissue Adds Independent Risks for Diastolic Dysfunction among Dialysis Patients. J. Magn. Reson. Imaging.

[13] Flüchter S., Haghi D., Dinter D., Heberlein W., Kühl H.P., Neff W., Sueselbeck T., Borggrefe M., Papavassiliu T. (2007). Volumetric Assessment of Epicardial Adipose Tissue with Cardiovascular Magnetic Resonance Imaging. Obesity.

[14] Iacobellis G., Barbaro G. (2019). Epicardial Adipose Tissue Feeding and Overfeeding the Heart. Nutrition.

[15] Patel V.B., Shah S., Verma S., Oudit G.Y. (2017). Epicardial Adipose Tissue as a Metabolic Transducer: Role in Heart Failure and Coronary Artery Disease. Heart Fail. Rev..

[16] Shmilovich H., Dey D., Cheng V.Y., Rajani R., Nakazato R., Otaki Y., Nakanishi R., Slomka P.J., Thomson L.E.J., Hayes S.W. (2011). Threshold for the Upper Normal Limit of Indexed Epicardial Fat Volume: Derivation in a Healthy Population and Validation in an Outcome-Based Study. Am. J. Cardiol..

[17] Iacobellis G., Bianco A.C. (2011). Epicardial adipose tissue: Emerging physiological, pathophysiological and clinical features. Trends in endocrinology and metabolism. Trends Endocrinol Metab..

[18] Ngo D.T., Gokce N. (2015). Epicardial Adipose Tissue: A Benign Consequence of Obesity?. Circ. Cardiovasc. Imaging.

[19] Eroğlu S. (2015). How Do We Measure Epicardial Adipose Tissue Thickness by Transthoracic Echocardiography?. Anatol. J. Cardiol..

[20] Sova M., Genzor S., Kolek V., Čtvrtlík F., Asswad A.G., Zela O., Tauber Z. (2018). Epicardial Fat in Patients with Chronic Obstructive Pulmonary Disease as a Marker of High Cardiovascular Risk-Review. Adv. Respir. Med..

[21] Mahabadi A.A., Lehmann N., Kälsch H., Bauer M., Dykun I., Kara K., Moebus S., Jöckel K.-H., Erbel R., Möhlenkamp S. (2014). Association of Epicardial Adipose Tissue and Left Atrial Size on Non-Contrast CT with Atrial Fibrillation: The Heinz Nixdorf Recall Study. Eur. Heart J. Cardiovasc. Imaging.

[22] Kaplan O., Kurtoglu E., Gozubuyuk G., Dogan C., Acar Z., EyupKoca F., Pekdemir H. (2015). Epicardial Adipose Tissue Thickness in Patients with Chronic Obstructive Pulmonary Disease Having Right Ventricular Systolic Dysfunction. Eur. Rev. Med. Pharmacol. Sci..

[23] Mahajan R., Kuklik P., Grover S., Brooks A.G., Wong C.X., Sanders P., Selvanayagam J.B. (2013). Cardiovascular Magnetic Resonance of Total and Atrial Pericardial Adipose Tissue: A Validation Study and Development of a 3 Dimensional Pericardial Adipose Tissue Model. J. Cardiovasc. Magn. Reson. Off. J. Soc. Cardiovasc. Magn. Reson..

[24] Wong C.X., Ganesan A.N., Selvanayagam J.B. (2017). Epicardial Fat and Atrial Fibrillation: Current Evidence, Potential Mechanisms, Clinical Implications, and Future Directions. Eur. Heart J..

[25] Saremi F., Mekhail S., Sefidbakht S., Thonar B., Malik S., Sarlaty T. (2011). Quantification of Epicardial Adipose Tissue: Correlation of Surface Area and Volume Measurements. Acad. Radiol..

[26] Park M.J., Jung J.I., Oh Y.S., Youn H.-J. (2010). Assessment of Epicardial Fat Volume with Threshold-Based 3-Dimensional Segmentation in CT: Comparison with the 2-Dimensional Short Axis-Based Method. Korean Circ. J..

[27] Szymański F.M. (2015). Epicardial adipose tissue in the pathogenesis of the cardiovascular disease-should we consider it as a cardiovascular risk factor and strive to reduce its amount?. Chor. Serca Naczyń.

[28] Chruściel P., Banach M. (2016). Atorvastatin in patients with overweight and obesity. Chor. Serca Naczyń.

[29] Ito T., Nasu K., Terashima M., Ehara M., Kinoshita Y., Ito T., Kimura M., Tanaka N., Habara M., Tsuchikane E. (2012). The Impact of Epicardial Fat Volume on Coronary Plaque Vulnerability: Insight from Optical Coherence Tomography Analysis. Eur. Heart J. Cardiovasc. Imaging.

[30] Yerramasu A., Dey D., Venuraju S., Anand D.V., Atwal S., Corder R., Berman D.S., Lahiri A. (2012). Increased Volume of Epicardial Fat Is an Independent Risk Factor for Accelerated Progression of Sub-Clinical Coronary Atherosclerosis. Atherosclerosis.

[31] Picard F.A., Gueret P., Laissy J.-P., Champagne S., Leclercq F., Carrié D., Juliard J.-M., Henry P., Niarra R., Chatellier G. (2014). Epicardial Adipose Tissue Thickness Correlates with the Presence and Severity of Angiographic Coronary Artery Disease in Stable Patients with Chest Pain. PLoS ONE.

[32] Nakanishi K., Fukuda S., Tanaka A., Otsuka K., Jissho S., Taguchi H., Yoshikawa J., Shimada K. (2014). Persistent Epicardial Adipose Tissue Accumulation Is Associated with Coronary Plaque Vulnerability and Future Acute Coronary Syndrome in Non-Obese Subjects with Coronary Artery Disease. Atherosclerosis.

[33] Kleinrok A., Głowa B. (2015). The Obesity and Its Meaning in Cardiovascular Diseases Part 1. Obesity as a Risk Factor. Med. Rev..

[34] Okada K., Ohshima S., Isobe S., Harada K., Hirashiki A., Funahashi H., Arai K., Hayashi D., Hayashi M., Ishii H. (2014). Epicardial Fat Volume Correlates with Severity of Coronary Artery Disease in Nonobese Patients. J. Cardiovasc. Med. Hagerstown Md.

[35] Demircelik M.B., Yilmaz O.C., Gurel O.M., Selcoki Y., Atar I.A., Bozkurt A., Akin K., Eryonucu B. (2014). Epicardial Adipose Tissue and Pericoronary Fat Thickness Measured with 64-Multidetector Computed Tomography: Potential Predictors of the Severity of Coronary Artery Disease. Clinics.

[36] Yamashita K., Yamamoto M.H., Igawa W., Ono M., Kido T., Ebara S., Okabe T., Saito S., Amemiya K., Isomura N. (2018). Association of Epicardial Adipose Tissue Volume and Total Coronary Plaque Burden in Patients with Coronary Artery Disease. Int. Heart J..

[37] Mazurek T. (2012). Nasierdziowa Tkanka Tłuszczowa a Przepływ w Tętnicach Wieńcowych. Czy Lokalizacja Ma Znaczenie?. Kardiol. Pol. Pol. Heart J..

[38] Cheng K.-H., Chu C.-S., Lee K.-T., Lin T.-H., Hsieh C.-C., Chiu C.-C., Voon W.-C., Sheu S.-H., Lai W.-T. (2008). Adipocytokines and Proinflammatory Mediators from Abdominal and Epicardial Adipose Tissue in Patients with Coronary Artery Disease. Int. J. Obes..

[39] Alexopoulos N., McLean D.S., Janik M., Arepalli C.D., Stillman A.E., Raggi P. (2010). Epicardial Adipose Tissue and Coronary Artery Plaque Characteristics. Atherosclerosis.

[40] Bertaso A.G., Bertol D., Duncan B.B., Foppa M. (2013). Epicardial Fat: Definition, Measurements and Systematic Review of Main Outcomes. Arq. Bras. Cardiol..

[41] Uygur B., Celik O., Ozturk D., Erturk M., Otcu H., Ustabasıoglu F.E., Yıldırım A. (2017). The Relationship between Location-Specific Epicardial Adipose Tissue Volume and Coronary Atherosclerotic Plaque Burden in Type 2 Diabetic Patients. Kardiol. Pol..

[42] Greif M., Becker A., von Ziegler F., Lebherz C., Lehrke M., Broedl U.C., Tittus J., Parhofer K., Becker C., Reiser M. (2009). Pericardial Adipose Tissue Determined by Dual Source CT Is a Risk Factor for Coronary Atherosclerosis. Arterioscler. Thromb. Vasc. Biol..

[43] Janik M., Hartlage G., Alexopoulos N., Mirzoyev Z., McLean D.S., Arepalli C.D., Chen Z., Stillman A.E., Raggi P. (2010). Epicardial Adipose Tissue Volume and Coronary Artery Calcium to Predict Myocardial Ischemia on Positron Emission Tomography-Computed Tomography Studies. J. Nucl. Cardiol. Off. Publ. Am. Soc. Nucl. Cardiol..

[44] Kalaycıoğlu E., Çetin M., Çinier G., Özyıldız A.G., Durmuş İ., Kırış T., Gökdeniz T. (2021). Epicardial Adipose Tissue Is Associated with Increased Systolic Pulmonary Artery Pressure in Patients with Chronic Obstructive Pulmonary Disease. Clin. Respir. J..

[45] Zagaceta J., Zulueta J.J., Bastarrika G., Colina I., Alcaide A.B., Campo A., Celli B.R., de Torres J.P. (2013). Epicardial Adipose Tissue in Patients with Chronic Obstructive Pulmonary Disease. PLoS ONE.

[46] Demir M., Acet H., Kaya H., Taylan M., Yüksel M., Yılmaz S., Sezgi C., Karadeniz G., Yenibertiz D. (2016). Relationship between Metabolic Syndrome and Epicardial Fat Tissue Thickness in Patients with Chronic Obstructive Pulmonary Disease. Anatol. J. Cardiol..

[47] Kiraz K., Gökdeniz T., Kalaycıoglu E., Börekçi A., Akyol S., Baykan A.O., Acele A., Karakoyun S., Seker T., Gür M. (2016). Epicardial Fat Thickness Is Associated with Severity of Disease in Patients with Chronic Obstructive Pulmonary Disease. Eur. Rev. Med. Pharmacol. Sci..

[48] Ding J., Hsu F.-C., Harris T.B., Liu Y., Kritchevsky S.B., Szklo M., Ouyang P., Espeland M.A., Lohman K.K., Criqui M.H. (2009). The Association of Pericardial Fat with Incident Coronary Heart Disease: The Multi-Ethnic Study of Atherosclerosis (MESA). Am. J. Clin. Nutr..

[49] van den Borst B., Gosker H.R., Schols A.M.W.J. (2013). Central Fat and Peripheral Muscle: Partners in Crime in Chronic Obstructive Pulmonary Disease. Am. J. Respir. Crit. Care Med..

[50] van den Borst B., Gosker H.R., Koster A., Yu B., Kritchevsky S.B., Liu Y., Meibohm B., Rice T.B., Shlipak M., Yende S. (2012). The Influence of Abdominal Visceral Fat on Inflammatory Pathways and Mortality Risk in Obstructive Lung Disease. Am. J. Clin. Nutr..

[51] Unubol M., Eryilmaz U., Guney E., Akgullu C., Kurt Omurlu I. (2014). Epicardial Adipose Tissue in Patients with Subclinical Hypothyroidism. Minerva Endocrinol..

[52] Sayin I., Erkan A.F., Ekici B., Kutuk U., Corakci A., Tore H.F. (2016). Thickening of the Epicardial Adipose Tissue Can Be Alleviated by Thyroid Hormone Replacement Therapy in Patients with Subclinical Hypothyroidism. Kardiol. Pol..

[53] Korkmaz L., Sahin S., Akyuz A.R., Ziyrek M., Anaforoglu I., Kose M., Erkan H., Ağaç M.T., Acar Z. (2013). Epicardial Adipose Tissue Increased in Patients with Newly Diagnosed Subclinical Hypothyroidism. Med. Princ. Pract. Int. J. Kuwait Univ. Health Sci. Cent..

[54] Asik M., Sahin S., Ozkul F., Anaforoglu I., Ayhan S., Karagol S., Gunes F., Algun E. (2013). Evaluation of Epicardial Fat Tissue Thickness in Patients with Hashimoto Thyroiditis. Clin. Endocrinol..

[55] Yazıcı D., Özben B., Toprak A., Yavuz D., Aydın H., Tarçın Ö., Deyneli O., Akalın S. (2015). Effects of Restoration of the Euthyroid State on Epicardial Adipose Tissue and Carotid Intima Media Thickness in Subclinical Hypothyroid Patients. Endocrine.

[56] Santos O.C., Silva N.A.O., Vaisman M., Turano M.D., Dytz M.G., Huber G.A., Braulio V.B., Teixeira P.F.S. (2015). Evaluation of Epicardial Fat Tissue Thickness as a Marker of Cardiovascular Risk in Patients with Subclinical Hypothyroidism. J. Endocrinol. Investig..

[57] Wójcik-Cichy K., Piekarska A. (2017). Influence of non-alcoholic fatty liver disease on risk of atherosclerosis and cardiovascular diseases. Hepatologia.

[58] Pisto P., Santaniemi M., Bloigu R., Ukkola O., Kesäniemi Y.A. (2014). Fatty Liver Predicts the Risk for Cardiovascular Events in Middle-Aged Population: A Population-Based Cohort Study. BMJ Open.

[59] Liu B., Li Y., Li Y., Liu Y., Yan Y., Luo A., Ren H., She Q. (2019). Association of Epicardial Adipose Tissue with Non-Alcoholic Fatty Liver Disease: A Meta-Analysis. Hepatol. Int..

[60] Fargion S., Porzio M., Fracanzani A.L. (2014). Nonalcoholic Fatty Liver Disease and Vascular Disease: State-of-the-Art. World J. Gastroenterol..

[61] Chen J., Mei Z., Yang Y., Dai C., Wang Y., Zeng R., Liu Q. (2022). Epicardial Adipose Tissue Is Associated with Higher Recurrence Risk after Catheter Ablation in Atrial Fibrillation Patients: A Systematic Review and Meta-Analysis. BMC Cardiovasc. Disord..

[62] Canpolat U., Aytemir K., Yorgun H., Asil S., Dural M., Özer N. (2016). The Impact of Echocardiographic Epicardial Fat Thickness on Outcomes of Cryoballoon-Based Atrial Fibrillation Ablation. Echocardiography.

[63] Chao T.-F., Hung C.-L., Tsao H.-M., Lin Y.-J., Yun C.-H., Lai Y.-H., Chang S.-L., Lo L.-W., Hu Y.-F., Tuan T.-C. (2013). Epicardial Adipose Tissue Thickness and Ablation Outcome of Atrial Fibrillation. PLoS ONE.

[64] Sanghai S.R., Sardana M., Hansra B., Lessard D.M., Dahlberg S.T., Aurigemma G.P., Fitzgibbons T.P., McManus D.D. (2018). Indexed Left Atrial Adipose Tissue Area Is Associated with Severity of Atrial Fibrillation and Atrial Fibrillation Recurrence among Patients Undergoing Catheter Ablation. Front. Cardiovasc. Med..

[65] Kawasaki M., Yamada T., Furukawa Y., Morita T., Tamaki S., Kida H., Sakata Y., Fukunami M. (2020). Are Cardiac Sympathetic Nerve Activity and Epicardial Adipose Tissue Associated with Atrial Fibrillation Recurrence after Catheter Ablation in Patients without Heart Failure?. Int. J. Cardiol..

[66] Conte M., Petraglia L., Poggio P., Valerio V., Cabaro S., Campana P., Comentale G., Attena E., Russo V., Pilato E. (2022). Inflammation and Cardiovascular Diseases in the Elderly: The Role of Epicardial Adipose Tissue. Front. Med..

[67] Guglielmi V., Maresca L., D’Adamo M., Di Roma M., Lanzillo C., Federici M., Lauro D., Preziosi P., Bellia A., Sbraccia P. (2014). Age-Related Different Relationships between Ectopic Adipose Tissues and Measures of Central Obesity in Sedentary Subjects. PLoS ONE.

[68] Karadag B., Ozulu B., Ozturk F.Y., Oztekin E., Sener N., Altuntas Y. (2011). Comparison of Epicardial Adipose Tissue (EAT) Thickness and Anthropometric Measurements in Metabolic Syndrome (MS) Cases above and under the Age of 65. Arch. Gerontol. Geriatr..

[69] Stramaglia G., Greco A., Guglielmi G., De Matthaeis A., Vendemiale G.L. (2010). Echocardiography and Dual-Energy X-ray Absorptiometry in the Elderly Patients with Metabolic Syndrome: A Comparison of Two Different Tecniques to Evaluate Visceral Fat Distribution. J. Nutr. Health Aging.

[70] Hell M.M., Ding X., Rubeaux M., Slomka P., Gransar H., Terzopoulos D., Hayes S., Marwan M., Achenbach S., Berman D.S. (2016). Epicardial Adipose Tissue Volume but Not Density Is an Independent Predictor for Myocardial Ischemia. J. Cardiovasc. Comput. Tomogr..

[71] Goeller M., Achenbach S., Marwan M., Doris M.K., Cadet S., Commandeur F., Chen X., Slomka P.J., Gransar H., Cao J.J. (2018). Epicardial Adipose Tissue Density and Volume Are Related to Subclinical Atherosclerosis, Inflammation and Major Adverse Cardiac Events in Asymptomatic Subjects. J. Cardiovasc. Comput. Tomogr..

[72] Nerlekar N., Thakur U., Lin A., Koh J.Q.S., Potter E., Liu D., Muthalaly R.G., Rashid H.N., Cameron J.D., Dey D. (2020). The Natural History of Epicardial Adipose Tissue Volume and Attenuation: A Long-Term Prospective Cohort Follow-up Study. Sci. Rep..

[73] Kataoka T., Harada K., Tanaka A., Onishi T., Matsunaga S., Funakubo H., Harada K., Nagao T., Shinoda N., Marui N. (2021). Relationship between Epicardial Adipose Tissue Volume and Coronary Artery Spasm. Int. J. Cardiol..

[74] Chen Y.-C., Lee W.-H., Lee M.-K., Hsu P.-C., Tsai W.-C., Chu C.-Y., Lee C.-S., Yen H.-W., Lin T.-H., Voon W.-C. (2020). Epicardial Adipose Tissue Thickness Is Not Associated with Adverse Cardiovascular Events in Patients Undergoing Haemodialysis. Sci. Rep..

[75] Nakazato R., Rajani R., Cheng V.Y., Shmilovich H., Nakanishi R., Otaki Y., Gransar H., Slomka P.J., Hayes S.W., Thomson L.E.J. (2012). Weight Change Modulates Epicardial Fat Burden: A 4-Year Serial Study with Non-Contrast Computed Tomography. Atherosclerosis.

[76] Fu C.-P., Sheu W.H.-H., Lee I.-T., Tsai I.-C., Lee W.-J., Liang K.-W., Lee W.-L., Lin S.-Y. (2013). Effects of Weight Loss on Epicardial Adipose Tissue Thickness and Its Relationship between Serum Soluble CD40 Ligand Levels in Obese Men. Clin. Chim. Acta Int. J. Clin. Chem..

[77] Willens H.J., Byers P., Chirinos J.A., Labrador E., Hare J.M., de Marchena E. (2007). Effects of Weight Loss after Bariatric Surgery on Epicardial Fat Measured Using Echocardiography. Am. J. Cardiol..

[78] Kim M.-K., Tomita T., Kim M.-J., Sasai H., Maeda S., Tanaka K. (2009). Aerobic Exercise Training Reduces Epicardial Fat in Obese Men. J. Appl. Physiol..

[79] Gaborit B., Jacquier A., Kober F., Abdesselam I., Cuisset T., Boullu-Ciocca S., Emungania O., Alessi M.-C., Clément K., Bernard M. (2012). Effects of Bariatric Surgery on Cardiac Ectopic Fat: Lesser Decrease in Epicardial Fat Compared to Visceral Fat Loss and No Change in Myocardial Triglyceride Content. J. Am. Coll. Cardiol..

[80] Parisi V., Petraglia L., D’Esposito V., Cabaro S., Rengo G., Caruso A., Grimaldi M.G., Baldascino F., De Bellis A., Vitale D. (2019). Statin Therapy Modulates Thickness and Inflammatory Profile of Human Epicardial Adipose Tissue. Int. J. Cardiol..

[81] Raggi P., Gadiyaram V., Zhang C., Chen Z., Lopaschuk G., Stillman A.E. (2019). Statins Reduce Epicardial Adipose Tissue Attenuation Independent of Lipid Lowering: A Potential Pleiotropic Effect. J. Am. Heart Assoc..

[82] Alexopoulos N., Melek B.H., Arepalli C.D., Hartlage G.-R., Chen Z., Kim S., Stillman A.E., Raggi P. (2013). Effect of Intensive versus Moderate Lipid-Lowering Therapy on Epicardial Adipose Tissue in Hyperlipidemic Post-Menopausal Women: A Substudy of the BELLES Trial (Beyond Endorsed Lipid Lowering with EBT Scanning). J. Am. Coll. Cardiol..

[83] Soucek F., Covassin N., Singh P., Ruzek L., Kara T., Suleiman M., Lerman A., Koestler C., Friedman P.A., Lopez-Jimenez F. (2015). Effects of Atorvastatin (80 Mg) Therapy on Quantity of Epicardial Adipose Tissue in Patients Undergoing Pulmonary Vein Isolation for Atrial Fibrillation. Am. J. Cardiol..

[84] Bouchi R., Terashima M., Sasahara Y., Asakawa M., Fukuda T., Takeuchi T., Nakano Y., Murakami M., Minami I., Izumiyama H. (2017). Luseogliflozin Reduces Epicardial Fat Accumulation in Patients with Type 2 Diabetes: A Pilot Study. Cardiovasc. Diabetol..

[85] Cosson E., Nguyen M.T., Rezgani I., Tatulashvili S., Sal M., Berkane N., Allard L., Brillet P.-Y., Bihan H. (2021). Epicardial Adipose Tissue Volume and Coronary Calcification among People Living with Diabetes: A Cross-Sectional Study. Cardiovasc. Diabetol..

[86] Abrishami A., Eslami V., Baharvand Z., Khalili N., Saghamanesh S., Zarei E., Sanei-Taheri M. (2021). Epicardial adipose tissue, inflammatory biomarkers and COVID-19: Is there a possible relationship?. Int. Immunopharmacol..

[87] Deng M., Qi Y., Deng L., Wang H., Xu Y., Li Z., Meng Z., Tang J., Dai Z. (2020). Obesity as a Potential Predictor of Disease Severity in Young COVID-19 Patients: A Retrospective Study. Obesity.

[88] Cereda A., Toselli M., Palmisano A., Vignale D., Khokhar A., Campo G., Bertini M., Loffi M., Andreini D., Pontone G. (2022). Coronary calcium score as a predictor of outcomes in the hypertensive Covid-19 population: Results from the Italian (S) Core-Covid-19 Registry. Hypertens. Res..

[89] Gasecka A., Pruc M., Kukula K., Gilis-Malinowska N., Filipiak K.J., Jaguszewski M.J., Szarpak L. (2021). Post-COVID-19 heart syndrome. Cardiol. J..

[90] Ryan P.M., Caplice N.M. (2020). Is Adipose Tissue a Reservoir for Viral Spread, Immune Activation, and Cytokine Amplification in Coronavirus Disease 2019?. Obesity.

[91] Lee M.-S., Duan L., Clare R., Hekimian A., Spencer H., Chen W. (2018). Comparison of Effects of Statin Use on Mortality in Patients with Heart Failure and Preserved Versus Reduced Left Ventricular Ejection Fraction. Am. J. Cardiol..

[92] Ahmadi N., Hajsadeghi F., Nabavi V., Arora R., Budoff M. (2013). The beneficial effects of statin therapy on epicardial adipose tissue and coronary plaque volumes with vulnerable characteristics measured by computed tomography angiographY. J. Am. Coll. Cardiol..

[93] Yamada Y., Takeuchi S., Yoneda M., Ito S., Sano Y., Nagasawa K., Matsuura N., Uchinaka A., Murohara T., Nagata K. (2017). Atorvastatin Reduces Cardiac and Adipose Tissue Inflammation in Rats with Metabolic Syndrome. Int. J. Cardiol..

[94] Tawakol A., Fayad Z.A., Mogg R., Alon A., Klimas M.T., Dansky H., Subramanian S.S., Abdelbaky A., Rudd J.H.F., Farkouh M.E. (2013). Intensification of Statin Therapy Results in a Rapid Reduction in Atherosclerotic Inflammation: Results of a Multicenter Fluorodeoxyglucose-Positron Emission Tomography/Computed Tomography Feasibility Study. J. Am. Coll. Cardiol..

[95] Konwerski M., Gąsecka A., Opolski G., Grabowski M., Mazurek T. (2022). Role of Epicardial Adipose Tissue in Cardiovascular Diseases: A Review. Biology.

[96] Varjabedian L., Bourji M., Pourafkari L., Nader N.D. (2018). Cardioprotection by Metformin: Beneficial Effects Beyond Glucose Reduction. Am. J. Cardiovasc. Drugs.

[97] Rabkin S.W., Campbell H. (2015). Comparison of Reducing Epicardial Fat by Exercise, Diet or Bariatric Surgery Weight Loss Strategies: A Systematic Review and Meta-Analysis. Obes. Rev..

